# Integrating genomic, transcriptomic, and phenotypic information to explore drug resistance in *Mycobacterium tuberculosis* sub-lineage 4.2.2.2

**DOI:** 10.1093/jambio/lxaf063

**Published:** 2025-03-12

**Authors:** Tesfaye Gebreyohannis Hailemariam, Abaysew Ayele, Tesfaye Gelanew, Abay Atnafu, Michael Brennan, Melaku Tilahun, Dawit Hailu Alemayehu, Zemedkun Abebe Debella, Yared Merid, Workineh Shibeshi, Abraham Aseffa, Kidist Bobosha, Yonas Hirutu, Simon J Waddell, Ephrem Engidawork

**Affiliations:** Department of Pharmacology and Clinical Pharmacy, School of Pharmacy, College of Health Science, Addis Ababa University, P.O.Box 9086, Addis Ababa, Ethiopia; Armauer Hansen Research Institute, P.O.Box 1005, Addis Ababa, Ethiopia; Armauer Hansen Research Institute, P.O.Box 1005, Addis Ababa, Ethiopia; Armauer Hansen Research Institute, P.O.Box 1005, Addis Ababa, Ethiopia; Armauer Hansen Research Institute, P.O.Box 1005, Addis Ababa, Ethiopia; Department of Global Health and Infection, Brighton and Sussex Medical School, University of Sussex, BN1 9PX, Brighton, United Kingdom; Armauer Hansen Research Institute, P.O.Box 1005, Addis Ababa, Ethiopia; Armauer Hansen Research Institute, P.O.Box 1005, Addis Ababa, Ethiopia; Addis Ababa Institute of Technology, Addis Ababa Univeristy, P.O. Box 1000, Addis Ababa, Ethiopia; Hawassa University College of Medicine and Health Sciences, P.O.Box 1560, Hawassa, Ethiopia; Department of Pharmacology and Clinical Pharmacy, School of Pharmacy, College of Health Science, Addis Ababa University, P.O.Box 9086, Addis Ababa, Ethiopia; Armauer Hansen Research Institute, P.O.Box 1005, Addis Ababa, Ethiopia; Armauer Hansen Research Institute, P.O.Box 1005, Addis Ababa, Ethiopia; Armauer Hansen Research Institute, P.O.Box 1005, Addis Ababa, Ethiopia; Department of Global Health and Infection, Brighton and Sussex Medical School, University of Sussex, BN1 9PX, Brighton, United Kingdom; Department of Pharmacology and Clinical Pharmacy, School of Pharmacy, College of Health Science, Addis Ababa University, P.O.Box 9086, Addis Ababa, Ethiopia

**Keywords:** *Mycobacterium tuberculosis*, lineage, drug-resistance, discordance, RNAseq, Ethiopia

## Abstract

**Aims:**

*Mycobacterium tuberculosis* (*Mtb*) remains a major global health challenge, particularly due to increasing drug resistance. Beyond the well-characterized mutations, the mechanisms involved in driving resistance appear to be more complex. This study investigated the differential gene expression of Ethiopian drug-resistant *Mtb* sub-lineage 4.2.2.2 clinical isolates through an integrated approach combining phenotypic, transcriptomic, and genomic analyses.

**Method and results:**

RNA sequencing was performed by isolating RNA from six *Mtb* strains (three drug-sensitive and three drug-resistant) during mid-logarithmic phase growth. Drug resistance was assessed through whole-genome analysis and phenotypic testing using the BACTEC Mycobacteria growth indicator tube (MGIT)™ 960 system. RNA profiling revealed significantly reduced expression of six genes: Rv0096, Rv2780, Rv3136, Rv3136A, Rv3137, and Rv3230c in drug-resistant isolates. These genes are not associated with known drug targets nor resistance mechanisms. Additionally, a discrepancy was noted between phenotypic resistance profiles and whole genome-based predictions, with the latter suggesting broader resistance. For instance, the missense mutation in rpoB p.Ser450Leu and katG p.Ser315Thr were identified with no change in phenotypic drug sensitivity to rifampicin and isoniazid, respectively.

**Conclusion:**

Identification of these differentially expressed genes and their networks could be useful in unraveling the complexities of *Mtb* drug resistance and in understanding the impact that drug resistance conferring mutations have on the physiology of drug-resistant *Mtb*.

Impact Statement The growing challenge of drug-resistant tuberculosis underscores the need for innovative approaches to understand and disable *Mtb* resistance mechanisms. This study highlights the complexities of drug resistance in Ethiopian *Mtb* sub-lineage 4.2.2.2 isolates. By integrating phenotypic, transcriptomic, and genomic analyses, it reveals reduced expression of six non-canonical resistance-associated genes, offering fresh insights into the molecular adaptations accompanying drug resistance. Moreover, the identified discrepancies between phenotypic resistance profiles and genome-based predictions could contribute evidence towards the utility of these methods for diagnostic applications. These findings advance our understanding of *Mtb* resistance mechanisms and pathways associated with drug resistance.

## Introduction

*Mycobacterium tuberculosis* (*Mtb*), the causative agent of tuberculosis (TB), is a historically significant pathogen that continues to pose a global public health challenge. TB affects millions of lives and remains one of the most lethal microorganisms worldwide (Goletti et al., 2025). Over the past two centuries, *Mtb* has been responsible for the deaths of approximately one billion individuals, underscoring its impact on human health (Torres Ortiz et al. , 2021). The *Mycobacterium tuberculosis* complex (MTBC) comprises a group of mycobacteria species that share 99.9% genetic similarity at the nucleotide level and possess identical 16S rRNA sequences (Brosch et al., 2002). Within the MTBC, there are ten lineages adapted to human hosts, identified as Lineages 1 through 10. Africa is the only continent where all MTBC lineages can be found (Cerezo-Cortés et al., 2022; Guyeux et al., 2024). Ethiopia is recognized as a major hub encompassing most of these lineages; according to a study by Mekonnen et al. (2019), the proportions of isolates for L4, L3, L1, and L7 were 62.3%, 21.7%, 7.9%, and 3.4%, respectively. Among the sub-lineages, the most prevalent clustered isolates were L4.2.2. ETH/SIT149 (L4.2.2.2), L4.10/SIT53, L3.ETH1/SIT25, and L4.6/SIT37, contributing 14.4%, 9.7%, 7.2%, and 5.5% of the total isolates, respectively. In northwest Ethiopia (from 2020 to 2022), the dominant drug-resistant sub-lineage was L4.2.2.ETH, which accounted for 34.5% of cases, representing 50% of drug-resistant isolates (Mekonnen et al., 2023). These findings highlight L4.2.2.2 as a sub-lineage marked by high transmissibility and drug resistance.

Drug resistance in *Mtb* presents a significant challenge to human health. *Mtb* has evolved resistance to most anti-TB drugs. The emergence of drug-resistant strains of *Mtb* threatens the effectiveness of disease control programs worldwide (Miotto et al., 2022). Treatment success rates on a global scale range from 85% for drug-sensitive TB to 56% for MDR-TB, further dropping to 39% for extensively drug-resistant tuberculosis (XDR-TB) (Wan et al. 2023). Although well-established canonical mutations are known to confer high-level drug resistance in *Mtb*, recent research indicates that *Mtb* can develop non-canonical resistance-associated mutations, which provide survival advantages in the presence of specific drugs and may serve as intermediates in the progression to high-level resistance (Martini et al., 2022). There is an urgent need for new drugs to shorten the duration of TB treatment and address the growing prevalence of infections caused by drug-resistant strains. However, selecting suitable targets for drug development remains challenging due to our limited understanding of how *Mtb* responds when target function is interrupted, or adapts to accommodate drug resistance conferring mutations.

Given the limited genetic diversity among *Mtb* strains, variations in *Mtb* phenotypes primarily stem from differences in the regulation of biochemical networks by key transcriptional regulators. By analyzing the transcriptomic profiles associated with specific genotypes, we can gain valuable insights into the underlying biology. This knowledge may help to identify potential targets for the development of novel drugs (Gomez-Gonzalez et al., 2019). In this study, we examined the expression of genes using RNA sequencing in *Mtb* sub lineage 4.2.2.2 strains isolated from Ethiopia. We compared drug-resistant with drug-sensitive isolates using *in vitro* phenotypic drug susceptibility testing (DST) and *in silico* predictions from whole genome sequencing (WGS).

## Materials and methods

### Study *M. tuberculosis* strains

This study utilized *Mtb* isolates obtained from active Ethiopian pulmonary TB patients participating in the TBGEN-Africa tuberculosis study. Six clinical isolates of *Mtb* sub-lineage 4.2.2.2 were obtained from various regions of Ethiopia, including Addis Ababa, Hawassa, Wolayita Sodo, and Gambella to include three drug susceptible and three drug resistant 4.2.2.2 isolates, which exhibited at least isoniazid (INH) resistance. These six clinically characterized *Mtb* isolates were further investigated using WGS.

### Growth of *M. tuberculosis* isolates *in vitro*

To generate the desired logarithmic-phase growth of bacilli, six clinical isolates of *Mtb* sub-lineage 4.2.2.2 were cultured to an optical density of 0.2 at 600 nm (OD600). These isolates were inoculated in a 1:4 ratio (32 ml total volume) using Middlebrook 7H9 broth, supplemented with 10% oleic acid, albumin, dextrose, catalase enrichment, 0.5% (v/v) glycerol, and 0.05% Tween 80, then incubated at 37°C for 11–15 days until they reached the mid-log phase of growth, with OD600 values ranging from 0.3 to 0.5.

### Phenotypic DST

The phenotypic drug susceptibility patterns of the isolates were determined using the BACTEC MGIT™ 960 system with the SIRE kit. Frozen isolates were thawed and subcultured in MGIT tubes. The day the MGIT system indicated a positive signal was recorded as day 0, tubes were incubated for one more day before commencing the DST. For signals detected on days 1 or 2, no dilution was required. However, for signals observed on days 3 to 5, a 1:5 dilution with sterile saline was performed prior to DST inoculation. If growth was detected after day 5, the samples were vortexed, diluted 1:100 with sterile saline, and 0.5 ml of the diluted sample was inoculated into a MGIT tube. The experiment was performed in accordance with the manufacturer's established standard operating procedures (Siddiqi and Rüsch-Gerdes 2006; Tilahun et al., 2023).

DST for first- and second-line drug resistance was conducted following the WHO technical manual (Siddiqi and Rüsch-Gerdes, 2006). The drug concentrations used were as follows: streptomycin (STM) 1.0 μg ml^−1^, INH 0.1 μg ml^−1^, rifampicin (RIF) 1.0 μg ml^−1^, ethambutol (EMB) 5.0 μg ml^−1^, bedaquiline 1.0 μg ml^−1^, delamanid 0.06 μg ml^−1^, moxifloxacin 0.25 μg ml^−1^, levofloxacin 1.0 μg ml^−1^, linezolid 1.0 μg ml^−1^, ofloxacin 2.0 μg ml^−1^, and clofazimine 1.0 μg ml^−1^. The DST process was marked as complete by the instrument when the growth control reached a growth unit (GU) value of 400. If the GU value was 400 or higher and the drug-containing tube had a reading below 100, the result was reported as “susceptible.” Conversely, if the GU value reached 400 and the drug-containing tube reading exceeded 100, the result was reported as “resistant.” Results were deemed invalid if the growth control GU value reached 400 in less than 4 days or failed to reach 400 within 21 days, triggering error messages X400 and X200, respectively. Quality control was ensured by testing each batch of MGIT medium and SIRE Kit with the pan-susceptible laboratory strain *M. tuberculosis* H37Rv (Tilahun et al., 2023).

### DNA extraction

For WGS of *Mtb*, high-quality DNA ranging from 0.5 to 5 µg with a concentration above 20 ng µl^−1^ was extracted using the Restricted fragment length polymorphism (RFLP) method. In brief, mycobacterial colonies grown on Lowenstein Jensen growth media were harvested into distilled water, followed by heat treatment and incubation with lysozyme, proteinase K, and NaCl/CTAB solutions. After DNA extraction with chloroform/isoamyl alcohol, the nucleic acids were precipitated with isopropanol and subsequently washed with ethanol. The DNA was dried, dissolved in molecular-grade water, and integrity assessed by agarose gel electrophoresis and Nanodrop spectrophotometer. DNA concentration was quantified using a Qubit fluorometer to ensure that the DNA met the necessary quality standards for sequencing (Dashti et al., 2009; Riaz et al., 2016).

### WGS bioinformatic analyses

The quality of the raw sequence reads was first evaluated using FASTQC (v0.12.1) (Yang et al., 2013). Low-quality reads, short fragments, and adapter sequences were then removed with Trimmomatic (v0.39) (Daniyarov et al., 2020), The remaining reads were subsequently mapped to the H37Rv reference genome (NC 000962.3) using BWA (v0.7.18) (Li, 2013). Single nucleotide polymorphisms (SNPs) were identified using the bcftools (v1.8) (Phelan et al., 2019). Drug resistance profiles and lineages were predicted with TBProfiler (v6.2.0) (Verboven et al., 2022). SNP based phylogenetic trees were constructed using IQ-REE (v2.0.3) with maximum likelihood model by replicating 1000 bootstraps value, and annotated and visualized using interactive Tree of Life (iTOL) (v6.9) (Yu 2020).

### Total RNA extraction

A 30-ml mid-log phase culture was transferred to 50 ml screw-cap tubes. The tubes were centrifuged at 2200 *g* at 4°C for 10 min to collect mycobacterial cells, discarding the supernatant. The resulting cell pellet was mixed with 1.2 ml TRIzol™ Reagent (Thermo Fisher Scientific, USA). The mixture was then transferred to a Lysing Matrix B tube (MP Biomedicals, Santa Ana, CA, USA) containing 0.1 mm silica beads. Bead beating was performed using a Fast Prep-24 5 G instrument (MP Biomedicals) at a speed setting of 6.5 for 45 s, followed by chloroform clean-up as previously described (Garton et al., 2008; Wildner et al., 2018).

Samples were purified and DNase-treated using RNeasy^®^ Mini Columns (Qiagen) following the manufacturer’s protocol. The purified RNA samples were quantified using the Nanodrop Spectrophotometer and RNA quality was assessed using the Agilent 4150 TapeStation system. Quality of the RNA samples was ensured by including samples with an RNA integrity number (RIN) >7 and an absorbance A260/A280 ratio between 1.8 and 2.1.

Ribosomal RNA was removed using the Illumina Ribo-Zero Plus Microbiome rRNA Depletion Kit (Illumina, USA) and libraries prepared for sequencing using TruSeq Stranded Total RNA kit designed for Illumina sequencing systems. Sequencing was performed using the Illumina NextSeq2000 platform, utilizing a P3 100-cycle protocol.

### RNAseq data analysis

FASTQC (v0.12.1) was used to visualize the quality of raw reads. Reads with low quality sequences, a Phred score less than 20 (Q < 20), and short fragments having <50 base-pairs were filtered out, and adapter sequences were trimmed using Fastp (v0.23.4) (Mohideen et al., 2020). Taxonomic identification and relative abundance of reads were assessed using Kraken software (Lu et al., 2017). Using the *M. tuberculosis* H37Rv (NC 000962.3) reference sequence obtained from the NCBI Database, clean reads were aligned and quantified using Hisat2 version 2.1.0 (Kim et al., 2019) and featureCounts version 2.0.6 (Liao et al., 2014), respectively. Subsequently, differential expression of genes was analyzed using DESeq2 package in R version 4.3.2 (Liu et al., 2021). Differentially expressed genes (DEGs) were identified, applying a cutoff threshold of log2 fold change > 1 comparing drug-resistant to drug-susceptible lineage 4.2.2.2 clinical isolates. *P*-values were adjusted for multiple comparisons using the Benjamini–Hochberg procedure with a significance cutoff *P* < 0.05 (Anders and Huber, 2012). Finally, Gene Ontology (GO) analysis, functional enrichment, and pathways analysis were conducted.

### Ethical clearance

Written informed consent was obtained from each patient prior to sample collection, ensuring their confidentiality and anonymity throughout the process. This study was approved by the Institutional Review Board of the College of Health Sciences, Addis Ababa University (Approval No. 072/21/Sop). This research is part of the TBGEN study—An integrated approach to unraveling susceptibility to tuberculosis in Africa, and received ethical approval from the Armauer Hansen Research Institute Institutional Ethics Review Committee (Approval No. P031/18).

## Results

### Discordance of phenotypic and whole genome-based drug resistance determination in *M. tuberculosis* clinical isolates

**Figure 1.**
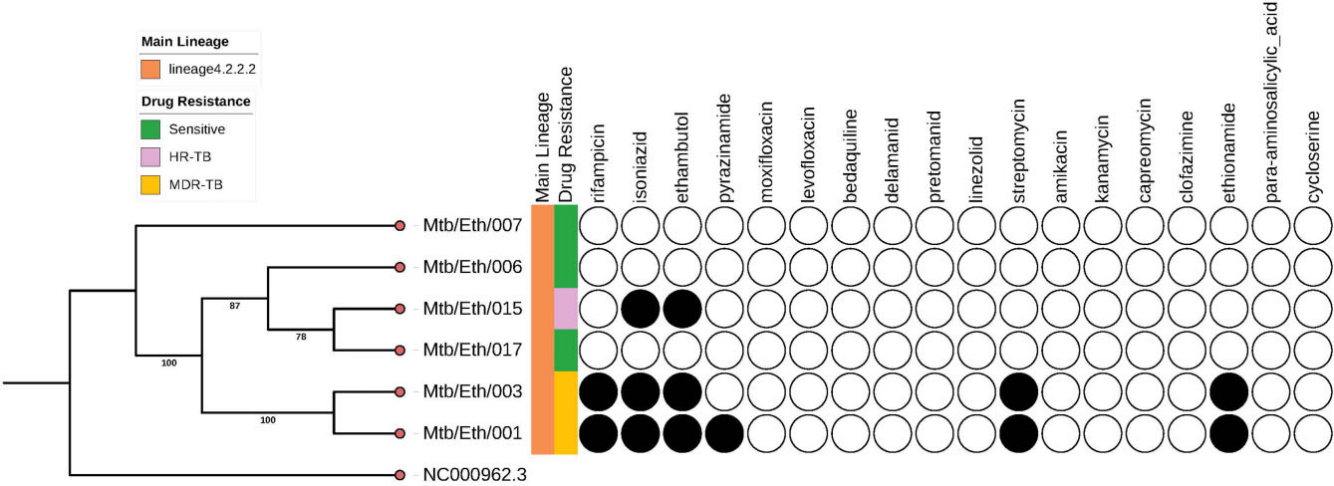
Phylogeny tree with drug susceptibility of *Mycobacterium tuberculosis* sub-lineage 4.2.2.2 isolates. NC 000962.3 is a Mtb H37Rv reference genome downloaded from NCBI. Shaded circles indicate a prediction of drug-resistance from the WGS analysis.

WGS was conducted on six *Mtb* sublineage 4.2.2.2 clinical isolates, including three drug-resistant and three drug-sensitive strains. From WGS analysis, two isolates (Mtb/Eth/001 and Mtb/Eth/003) were predicted as MDR (INH and RIF resistance), while one isolate (Mtb/Eth/015) was predicted to be resistant to INH and EMB (Fig. 1). All three resistant strains shared the same INH resistance conferring katG p.Ser315Thr mutation (Table 1). Additionally, the two isolates (Mtb/Eth/001 and Mtb/Eth/003) resistant to ethionamide carried an identical ethA p.Met1 mutation (loss of start codon). WGS analysis revealed distinct mutations associated with resistance to RIF in the two MDR-TB isolates (Table 1). Interestingly, phenotypic drug sensitivity testing indicated discrepancies, identifying only one isolate as RIF resistant and therefore MDR (isolate Mtb/Eth/001) as the WGS predicted RIF and STM resistance was not observed in phenotypic DST for isolate Mtb/Eth/003 (Table 2).

**Table 1.** Whole genome-based drug resistance prediction with corresponding mutation for the selected *Mycobacterium tuberculosis* sub-lineages 4.2.2.2.

Samples	Drug resistance type	Number of drug-resistant variants	Number of other variants	Rifampicin	Isoniazid	Ethambutol	Pyrazinamide	Ethionamide	Streptomycin
**Mtb/Eth/003**	MDR-TB	6	25	rpoB p.His445Cys	katG p.Ser315Thr	embA c.-16C > T embB p.Met306Ile		ethA p.Met1?	
**Mtb/Eth/001**	MDR-TB	8	58	rpoB p.Ser450Leu	katG p.Ser315Thr	embB p.Asp1024Asn embB p.Gly406Ala	pncA p.Val130Gly	ethA p.Met1?	Gid p.Gly69Asp rpsL p.Lys88Thr
**Mtb/Eth/015**	HR-TB	2	32	-	katG p.Ser315Thr	embB p.Met306Ile	-	-	
**Mtb/Eth/017**	Sensitive	0	24	-	-	-	-	-	
**Mtb/Eth/007**	Sensitive	0	24	-	-	-	-	-	
**Mtb/Eth/006**	Sensitive	0	24	-	-	-	-	-	

Eth. Ethionamide; HR, isoniazid resistance; MDR-TB, multidrug resistance, tuberculosis; Mtb, *Mycobacterium tuberculosis*.

**Table 2.** Phenotypic drug sensitivity of *Mycobacterium tuberculosis* 4.2.2.2 sub-lineage isolates.

Isolate identifier	Isoniazid	Rifampicin	Ethambutol	Streptomycin	Second line Anti TB Drugs
Mtb_Eth_006	Sensitive	Sensitive	Sensitive	Sensitive	Sensitive
Mtb_Eth_007	Sensitive	Sensitive	Sensitive	Sensitive	Sensitive
Mtb_Eth_017	Sensitive	Sensitive	Sensitive	Sensitive	Sensitive
Mtb_Eth_001	Resistance	Resistance	Sensitive*	Resistance	Sensitive
Mtb_Eth_003	Resistance	Sensitive*	Sensitive*	Sensitive	Sensitive
Mtb_Eth_015	Resistance	Sensitive	Sensitive*	Sensitive	Sensitive

Asterisk (*) marks discrepancies between phenotypic DST results and genotypic WGS predictions. The second-line anti-TB drugs analyzed in this phenotypic study include bedaquiline, delamanid, moxifloxacin, levofloxacin, linezolid, ofloxacin, and clofazimine.

### Transcriptomic profiles of resistant and sensitive *M. tuberculosis*

**Figure 2.**
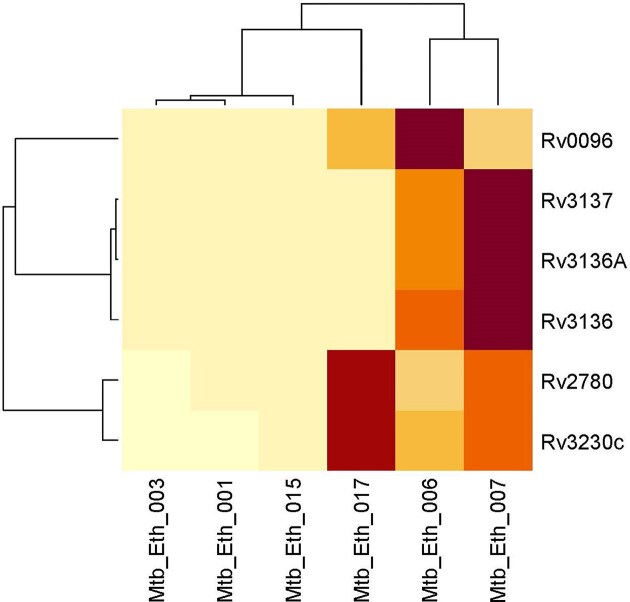
Clustered heatmap of significantly differentially expressed genes between drug resistant and drug-sensitive *Mycobacterium tuberculosis* lineage 4.2.2.2 isolates. The heat map displays the expression levels of genes across different isolates, with rows representing individual genes and columns representing clinical isolates. The color intensity indicates expression levels, with light yellow signifying lower expression and dark red indicating higher expression. The dendrograms on the top and left show hierarchical clustering, grouping genes and samples based on their expression similarities.

**Figure 3.**
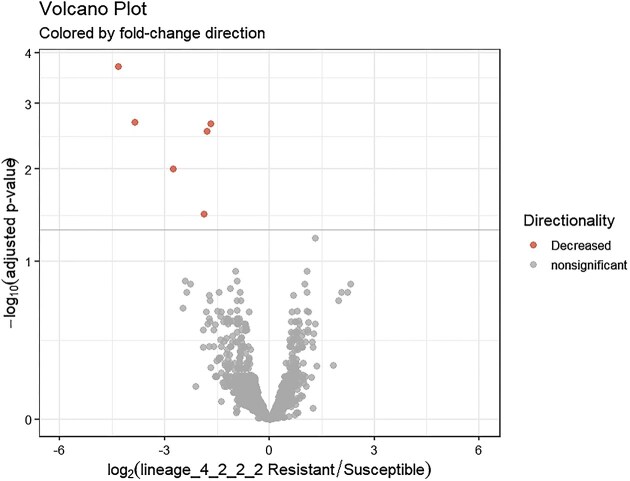
Volcano plot illustrating differentially expressed genes between drug-resistant and drug-sensitive *Mtb* sub-lineage 4.2.2.2 isolates. The *x*-axis represents the log2 fold change and the *y*-axis shows the −log10 *P*-values. Negative values indicate downregulation in drug-resistant relative to drug sensitive isolates. Red coloring denotes significantly downregulated genes with >1 log2 fold change and <0.05 adjusted *P*-value.

Following quality control and trimming procedures, ~12 to 16 million single-ended reads for each sample were aligned to the *M. tuberculosis* (H37Rv) reference genome, with an average alignment rate of 85.26%. Differential expression analysis revealed six genes that were differentially regulated in drug-resistant compared to the drug-sensitive isolates. All the six genes: Rv0096, Rv2780, Rv3136, Rv3136A, Rv3137, and Rv3230c were downregulated in the drug-resistant group. These DEGs were visualized using a clustered heat map and volcano plot as depicted in Figs 2 and 3. The DEGs were classified according to functional categories as detailed in Table 3.

**Table 3.** Differentially expressed genes in drug resistant *mycobacterium tuberculosis* isolates compared to drug sensitive. the table details log2 fold change (relative to drug sensitive isolates), *P*-value adjusted for multiple testing comparison and functional categories.

Rv Number	Base Mean	log2Fold Change	*P* values	Adjusted *P* values	Functional categories of genes
Rv0096	180.49	-1.88	5.21e-05	0.0343	PPE family protein PPE1
Rv2780	1612.69	-1.80	2.94e-06	0.0029	L-alanine dehydrogenase Ald
Rv3136	1925.37	-3.85	1.05e-06	0.0021	Conserved protein
Rv3136A	785.90	-4.32	5.06e-08	0.0002	Conserved protein
Rv3137	543.09	-2.75	1.25e-05	0.0099	Probable monophosphatase
Rv3230c	961.09	-1.69	1.66e-06	0.0022	Hypothetical oxidoreductase

## Discussion

TB poses a significant global health challenge, and the emergence of drug-resistant *Mtb* strains further complicates control efforts. Unlike some bacteria, *Mtb* does not gain drug resistance through horizontal gene transfer but by the accumulation of drug-resistance conferring chromosomal mutations. A deeper understanding of drug resistance mechanisms is crucial for developing drugs that can overcome existing resistance or target alternative biochemical pathways. This knowledge will not only help prevent the emergence of drug resistance but also identify cellular components that impact the action of anti-TB drugs.

A missense mutation in rpoB p.Ser450Leu aligns with phenotypic drug resistance in MDR isolate Mtb/Eth/001, whereas the rpoB p.His445Cys missense mutation did not confer phenotypic resistance to rifampicin in isolate Mtb/Eth/003 in our hands. The rpoB p.Ser450Leu variant has been documented in the WHO Catalogue of Mutations in the *M. tuberculosis* complex and their association with drug resistance, demonstrating a sensitivity of 64.4% and a specificity of 99.3% for detecting rifampicin-resistant phenotypes. However, rpoB p.His445Cys variant has a sensitivity of 0.4% and a specificity of 100% for detecting rifampicin-resistant phenotypes (World Health Organization, 2023). The substitution of the polar amino acid serine with the non-polar leucine at position 450 may disrupt rifampicin binding to rpoB, leading to drug resistance (Go and Miyazawa, 1980). No additional variants or SNP differences were detected in the rpoB gene between the two clinical isolates, both of which were classified as MDR-TB based on WGS analysis. The katG p.Ser315Thr missense mutation was associated with phenotypic drug resistance in all drug resistant isolates and is also reported in WHO Catalogue with a sensitivity of 77.8% and a specificity of 99.1% for detecting INH-resistant bacilli (World Health Organization, 2023).

Our phenotypic drug sensitivity results indicate susceptibility to EMB (Mtb/Eth/001 and Mtb/Eth/003), whereas WGS-based drug sensitivity analysis identified resistance conferring mutations, with different mutations occurring at various positions for different samples. In line with our finding, routine phenotypic analysis failed to detect EMB resistance in 91.4% of resistant isolates, highlighting the challenges of EMB phenotypic testing. The inability of culture-based methods to reliably identify true EMB resistance negatively impacts TB control programs. However, a significant proportion of phenotypically EMB-resistant isolates (~30%) still lack identifiable mutations in *embB*, underscoring the need for a comprehensive understanding of EMB resistance mechanisms in clinical isolates (Johnson et al., 2006). Differences between molecular and phenotypic ethambutol resistance results are likely due to limitations in conventional susceptibility testing methods (Plinke et al., 2009). Our study found higher drug resistance predicted through WGS compared to phenotypic drug sensitivity testing. This discrepancy may stem from the difficulties in generating accurate and reproducible drug sensitivity data from pathogenic clinical isolates in the laboratory. Given these discrepancies, an improved and an integrative approach combining phenotypic and genotypic methods is essential for accurately characterizing drug resistance and optimizing TB treatment strategies.

By analyzing gene expression levels and genetic regions associated with differential expression, we identified six DEGs in our *Mtb* sub-lineage 4.2.2.2 drug-resistant isolates that may be linked to the acquisition of or adaptation to drug resistance. Rv0096, Rv2780, Rv3136, Rv3136A, Rv3137 and Rv3230c were downregulated in the drug-resistant isolates compared to drug sensitive *Mtb* sub-lineage 4.2.2.2. These genes are not direct targets of the current anti-TB drugs. Additionally, no common SNPs were observed in the six DEGs in the drug-resistant group compared to the drug sensitive group.

The Rv0096 (PPE1) gene encodes a putative member of the PPE (proline-glutamate and proline-proline-glutamate) family and is part of the rv0096–rv0101 operon, which has been shown to produce a virulence-associated lipopeptide (Sao Emani and Reiling, 2024). This operon encodes enzymes responsible for synthesizing an isonitrile lipopeptide, potentially involved in biofilm formation and copper acquisition under copper-limited conditions. Recent studies suggest that PPE1 is a functional component of this cluster, highlighting its co-essentiality with the other genes in the operon (Jinich et al., 2022). Consistent with our findings, downregulation of Rv0096 has been reported in rifampicin-resistant *Mtb* H37Rv strains carrying the H526Y mutation in the rpoB gene, but not in rifampicin-susceptible H37Rv wild-type strains (de Knegt et al., 2013). A loss-of-function mutation in Rv0096 was also found to confer resistance to D-cycloserine in MDR *Mtb* (Sao Emani and Reiling, 2024).

Rv2780, also downregulated in drug-resistant isolates, encodes L-alanine dehydrogenase (*ald*), an enzyme catalyzing the oxidative deamination of L-alanine to pyruvate, which is subsequently utilized in peptidoglycan synthesis (Sao Emani and Reiling, 2024). Rv2780 is also shown to catalyze the reductive amination of glyoxylate to glycine and is upregulated under conditions such as hypoxia, nutrient starvation, and when alanine serves as the sole nitrogen source, suggesting multiple physiological roles, particularly in carbon and nitrogen metabolism (Sao Emani and Reiling, 2024). Desjardins et al. (2016) showed that deletion of *ald* increased *Mtb* resistance to D-cycloserine and reintroducing the gene partially restored susceptibility to the drug. L-alanine treatment effectively inhibited bacterial survival, comparable to RIF, and a combination of L-alanine with RIF further enhanced bacterial clearance, suggesting L-alanine as a promising adjunct to TB therapy. The small-molecule and Rv2780 inhibitor (S)-N-(5-(3-fluorobenzyl)-1H-1,2,4-triazol-3-yl) tetrahydrofuran-2-carboxamide (GWP-042) significantly reduced the survival of both *Mtb* H37Rv and multidrug-resistant (MDR-TB) strains.

This study also revealed that the cluster of genes Rv3136, Rv3136A, and Rv3137 were repressed in drug-resistant relative to drug-sensitive sub-lineage 4.2.2.2 isolates. Rv3136 encodes the PPE51 protein, which has been implicated in *Mtb*’s ability to uptake disaccharides. Nonsynonymous mutations in Rv3136 were resistant to high concentrations of thio-disaccharides, known for their bactericidal effect on *Mtb*, restoring with a functional gene reinstated their sensitivity to the wild type level (Korycka-Machała et al., 2020). Our study also observed a downregulation of Rv3135 (*ppe50*), with a log fold change of 2, though the difference was not statistically significant (Supplementary Table 1). The ppe50-ppe51 operon is critical for INH and RIF tolerance phenotypes in RNase J (Rv2752c) mutant *Mtb* strains. Deleting this operon induced drug tolerance and overexpression restored drug sensitivity, highlighting its role in drug susceptibility (Martini et al., 2022). Additionally, a study identified a crucial role of *ppe50-ppe51* mutants in enhancing survival and contributing to the development of resistance (Bellerose, 2020). Our study also demonstrated reduced expression of the Rv3136A gene, encoding a conserved 110-amino-acid protein of unknown function. Rv3137, a member of the inositol monophosphatase (IMPase) gene family was also downregulated. This gene functions as the *Mtb* histidinol phosphate phosphatase (HolPase), specifically catalyzing the dephosphorylation of histidinol phosphate (Jha et al., 2018). *Mtb* has developed strategies to acquire amino acids from host cells, while also maintaining some *de novo* biosynthesis pathways. In response, the host mounts an immune defense by upregulating histidine-catabolizing enzymes through interferon gamma (IFN-γ)-mediated signaling, aiming to deprive the bacillus of intracellular free histidine. *Mtb* circumvents this immune response by synthesizing histidine *de novo*, and histidine auxotrophs are unable to proliferate (Dwivedy et al., 2021). Studies have demonstrated an association between INH resistance and reduced expression of Rv3137 in INH-resistant groups compared to susceptible groups (Mehaffy et al., 2018) (Martini et al., 2022). Rv3137 is also downregulated during exponential phase in the highly successful *Mtb* MtZ strain from Aragon, Spain (Comín et al. 2023). The observed downregulation of Rv3137 in highly successful *Mtb* strains as well as in drug-resistant isolates aligns with our findings and perhaps highlights the role of this gene in pathogenesis and drug resistance.

Rv3230c, also repressed in drug-resistant isolates, encodes an oxidoreductase that forms a functional complex with the integral membrane stearoyl-CoA desaturase DesA3 to convert saturated stearic acid into unsaturated oleic acid, utilizing molecular oxygen and NADPH in the process. Oleic acid is critical for maintaining membrane composition and physiological functions by regulating membrane fluidity through the incorporation of unsaturated fatty acids into membrane lipids. Oleic acid is thought to serve as a precursor in mycolic acid synthesis, contributing to the introduction of double bonds in specific mycolic acid structures. This suggests that DesA3 and Rv3230c are likely essential for lipid metabolism, playing key roles in the formation of both the cell membrane and cell wall in *Mtb* (Rehberg et al., 2019). A genomic study conducted on the clonal expansion of XDR-TB of the rare Proto-Beijing genotype identified a 1 285 bp deletion within the desA3 and oxidoreductase Rv3230c genes (Srilohasin et al., 2020). Furthermore, a WGS study conducted in Hanoi revealed a significant association between a 1-bp deletion in Rv3230c and clustered strains carrying the katG-S315T mutation, a key marker of INH resistance among the population (Hang et al., 2019).

A gene network analysis revealed that alternative sigma factors may regulate the downregulated genes. Specifically, sigma M (SigM) suppresses the expression of Rv3136 and Rv3137 while also modulating the regulation of Rv0096 (Raman et al., 2006). As a member of the extracytoplasmic function subfamily of alternative sigma factors, *Mtb* SigM is expressed at low levels *in vitro* and does not appear to play a role in stress response regulation. Instead, SigM positively regulates genes involved in the synthesis of surface or secreted molecules. Its role in repressing virulence-associated surface lipids while upregulating Esx family secreted proteins and nonribosomal peptide synthetase genes suggests that SigM may be involved in long-term adaptation to specific host environments during infection (Raman et al., 2006). The regulation of three of six genes by SigM suggests that it may play a role in the adaptation to drug resistance in lineage 4.2.2.2. Additionally, Rv3230c is negatively regulated by the alternative sigma factor SigD, encoded by Rv3414c (Raman et al., 2004). Further research should prioritize exploring the mechanisms underlying resistance to anti-TB drugs, including pathways differentially regulated in drug resistant isolates that may enable bacilli to adjust to the impact that drug-conferring mutations may have on *Mtb* metabolism.

Although this study offers insights into drug resistance mechanisms in *Mtb*, it has limitations. One notable limitation of the research lies in the small sample size, which can be attributed to the difficulties in obtaining suitable samples that adequately represent different drug resistance profiles in preferred *Mtb* sub-lineages.

Overall, our analysis of *Mtb* sub-lineage 4.2.2.2 clinical isolates comparing different drug resistance profiles to drug sensitive *Mtb*, revealed six common DEGs (Rv0096, Rv2780, Rv3136, Rv3136A, Rv3137, and Rv3230c) associated with drug resistance. The regulation of these genes was not directly linked to primary drug targets, nor impacted by common chromosomal SNPs. These findings highlight the potential of integrating phenotypic, genomic, and transcriptomic data to enhance our understanding of drug resistance mechanisms and identify promising drug targets.

## Supplementary Material

lxaf063_Supplemental_Files

## Data Availability

The raw data supporting the conclusions of this article will be made available by the authors on reasonable request.
